# Prolonged normothermic perfusion: a promising approach to kidney transplantation

**DOI:** 10.1093/ckj/sfaf210

**Published:** 2025-07-03

**Authors:** Michele F Eisenga, Rainer Oberbauer

**Affiliations:** Department of Internal Medicine, Division of Nephrology, University of Groningen, University Medical Center Groningen, Groningen, The Netherlands; Department of Nephrology, Medical University of Vienna, Vienna, Austria

In the 17 May 2025 issue of *Nature Communications*, Richard Dumbill and colleagues from Oxford, Coventry and Innsbruck reported a single-center Phase 1 study involving 36 patients who received deceased donor kidney transplants (44% donation after circulatory death). These kidneys were maintained on normothermic machine perfusion (NMP) for a median duration of approximately 6 h (range 2.2–23.4) [1]. The control group consisted of historical transplants (*n* = 72), which were matched 2:1 based on four variables—donor type, the 2019 UK Kidney Donor Risk Index, induction immunosuppression and the cold ischemic time prior to NMP.

**Figure 1:**
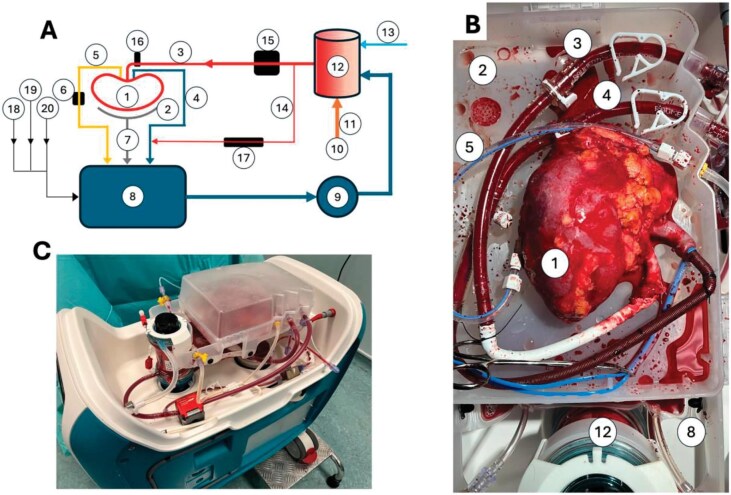
Scheme of the normothermic machine perfusion and device from OrganOx Ltd in use. (A) schematic overview; (B) kidney during perfusion; (C) machine with kidney on-board. From https://www.nature.com/articles/s41467-025-59829-5.

The primary outcome was death-censored graft survival at 1 month. The secondary objectives included ischemia–reperfusion injury severity determined by biomarkers at 2 h (such as NGAL, GST-Pi, LDH, L-FABP and IL-18), as well as vascular resistance and the technical performance of the perfusion device. The study was sponsored by OrganOx Ltd, Oxford, which provided the perfusion device (Fig. 1); four authors reported affiliations with the company.

All kidneys were suitable for perfusion regardless of vascular anatomy: 27 of 36 kidneys had a single artery, 8 had two renal arteries and 1 had three renal arteries. One perfused kidney was discarded due to abnormally elevated vascular resistance.

Induction immunosuppression was administered using alemtuzumab in 72% of cases and basiliximab in 28%. The cold ischemic time prior to perfusion was approximately 8.5 h in both cases and controls. NMP added approximately 7 h of total preservation time. The incidence of delayed graft function (DGF) was 36% and 38% in the groups, respectively. The incidence of functional DGF (fDGF), defined as less than a 10% creatinine reduction on each of the first 3 days, was 50% in the study group but 61% in the control group.

The main findings of the study indicated that all 36 transplanted organs were functioning at 1 month (primary endpoint). As per the study design, the matched control group exhibited similar outcome data but demonstrated a numerically higher incidence of functional DGF. Within the first year, one biopsy-confirmed acute rejection (BCAR) occurred, one graft was lost and one patient died. The estimated glomerular filtration rate (eGFR) at both 3 and 12 months was approximately 46 mL/min. Furthermore, a statistically significant correlation was observed between most *ex situ* biomarkers and graft function at 1 year; for example, the explained variability in eGFR at 12 months attributable to delta GST-Pi was nearly 30%.

The paper includes a wealth of additional data, which are beyond the scope of this brief in-context summary. Based on the reported findings, the authors concluded that NMP is useful for facilitating the logistics of deceased donor kidney transplantation and serves as a promising organ assessment technique. Furthermore, NMP may have the potential to expand the donor pool, enabling the transplantation of kidneys that would otherwise be discarded. We fully agree that prolonged *ex vivo* storage of transplant kidneys without compromising quality would represent a major advantage for transplant centers and organ procurement organizations, allowing planned transplant surgeries during daytime hours by a rested team and better preparation of recipients. Evaluating organ quality *ex vivo* over time could allow for more liberal acceptance of marginal organs and may even become a critical advancement for the transplantation of life-sustaining organs. As the authors suggest, an important aspect to evaluate in the future is whether transporting the NMP to the donor site and thereby eliminating the initial period of cold ischemia could lead to improved outcomes.

In a recent large randomized controlled trial involving 338 transplant recipients who underwent 1 h of NMP following cold storage, Hosgood *et al.* also did not observe a difference in the incidence of DGF (approximately 60% in each group) as the primary outcome. However, they demonstrated that NMP was both feasible and safe [2]. Recently published pig kidney autotransplantation models with NMP are, for various reasons, not directly comparable to the human clinical setting [3].

In context, this represents an elegant and well-performed Phase 1 study of NMP in kidney transplantation. What this study clearly demonstrates is that prolonged NMP with total ischemic times exceeding 1  day is both feasible and safe. These findings are encouraging and will likely stimulate further investigation in the highly competitive field of organ preservation.
